# Hidden complexity in the ontogeny of sexual size dimorphism in male-larger beetles

**DOI:** 10.1038/s41598-018-24047-1

**Published:** 2018-04-12

**Authors:** Tomáš Vendl, Petr Šípek, Ondřej Kouklík, Lukáš Kratochvíl

**Affiliations:** 1 Department of Zoology, Faculty of Science, Charles University, Viničná 7, 12844 Praha 2, Czech Republic; 2 Department of Ecology, Faculty of Science, Charles University, Viničná 7, 12844 Praha 2, Czech Republic; 3 Crop Research Institute, Drnovská 507, 16106 Praha 6 - Ruzyně, Czech Republic

## Abstract

Sexual size dimorphism (SSD) is widespread among animals, but its developmental mechanisms are not fully undestood. We investigated the proximate causes of SSD in three male-larger and one monomorphic scarab beetles using detailed monitoring of growth in individual instars. Apart from the finding that SSD in all three male-larger species started to develop already in the first larval instar, we generally found a high variability in SSD formation among the species as well as among instars. Overall, sexual differences in developmental time, average growth rate, as well as in the shape of the growth trajectory seem to be the mechanisms responsible for SSD ontogeny in scarab beetles. In the third instar, when the larvae attain most of their mass, the males had a similar or even lower *instantaneous* growth rate than females and SSD largely developed as a consequence of a longer period of rapid growth in males even in cases when the sexes did not differ in the total duration of this instar. Our results demonstrate that a detailed approach, examining not only the average growth rate and developmental time, but also the shape of the growth trajectory, is necessary to elucidate the complex development of SSD.

## Introduction

Female-biased sexual size dimorphism (SSD), i.e. situation when females are the larger sex, is prevalent in poikilothermic vertebrates^1,2^ and most arthropods, including insects^3,4^. Stillwell *et al*.^4^ concluded that only 4–10% of studied insect species exhibit male-biased SSD, while Teder^5^ estimated its frequency in insects at 16%. Accordingly, most studies dealing with the development of SSD in insects are focused on species with a female-biased SSD, but data on cases where males are the larger sex are scarce^6^.

In spite of the importance and wide occurrence of SSD, the developmental processes leading to adult differences between sexes are still only relatively poorly understood. In insects, there is variability in *when* SSD arises during ontogeny. There is evidence that the onset of sexual differences in size occurs early in development^7–9^, whereas other studies documented a rise of SSD only much later in the development^10–12^. Moreover, the time when the sexes start to depart in growth may depend on environmental conditions^8^.

Similarly to *when*, the question *how* SSD arises does not have a conclusive answer in insects yet. Sexual differences in developmental time connected with different time for body increase^7,13,14^, in growth rate^8,15–17^ or in both of these variables^18–21^ have been demonstrated repeatedly as proximate mechanisms of SSD development. Generally, it appears that sex-specific growth rate is the prevalent mechanism of SSD in insects^6^, although other studies suggest that differences in developmental time contribute significantly to SSD development as well^5,22^. Moreover, differential mass loss at eclosion^9,23^ and a different number of larval instars between sexes^10^ have been documented as other ontogenetic mechanisms leading to SSD in insects. On the other hand, sexual differences in egg or hatchling size are rather uncommon in this group^7,19,24^, but see^25^.

Nevertheless, as was emphasized by Tammaru *et al*.^7^, many previous studies on the development of SSD in insects are not sufficiently detailed because they do not take into account the entire complexity of insect growth and development, mainly the presence of separate larval instars. Particular instars may differ in physiology^26,27^, as well as in growth patterns^12,28^. Growth typically stops before moulting in each instar^29,30^. Growth trajectories of holometabolous insects are typically not linear but rather sigmoidal^31,32^, well described by asymptotic curves, which is also true for scarab beetles^12,30^. This holds especially for their final larval instar, where the variability in the duration of a period of very slow growth contributes to inter-, as well as intra-instar differences in body size and developmental time. In the case of holometabolous insects, the *de novo* development of adult organs from imaginal discs takes place mainly during the final larval instar and in the pupa. Therefore, it is reasonable to assume that there may be differences in growth between the last and the previous larval instars as well as between sexes in the last instar^24,33^. For all these reasons, investigation of the ontogeny of SSD may be incomplete and inaccurate if larval development is monitored integrally (for example, if growth rate is expressed simply as adult size divided by egg-to-adult development time), therefore a correct identification of the proximate causes of SSD requires continuous recording of larval growth^7^.

In this study, we explore the ontogeny of SSD in three male-larger scarab beetles, the rose chafers *Eudicella gralli* (Buquet, 1836) and *Dicronorhina micans* (Drury, 1773; Cetoniinae) and the rhinoceros beetle *Xylotrupes gideon* (Linnaeus, 1767; Dynastinae). For comparison, we followed growth patterns in a non-dimorphic species of rose chafer, *Oxythyrea pantherina* (Gory & Percheron, 1833). Rose chafers and rhinoceros beetles represent closely related groups of the phytophagous lineage of Scarabaeidae^34^, a clade with prevalent sexual dimorphism, where males often possess cephalic and/or pronotal horns, prolonged forelimbs, or other excessive structures^35^. These structures are used as weapons in male–male combats^36^. This intrasexual selection is probably the selective agent responsible for male-biased SSD in these beetles. Based on regular, detailed and instar-specific recording of body mass from hatching to maturity, we examined the development of sexual differences in body size. Specifically, we tested sexual differences in developmental time, growth rate, and the shape of growth trajectories of individual instars to reveal the detailed ontogeny of SSD.

## Methods

### Studied species

**Figure 1:**
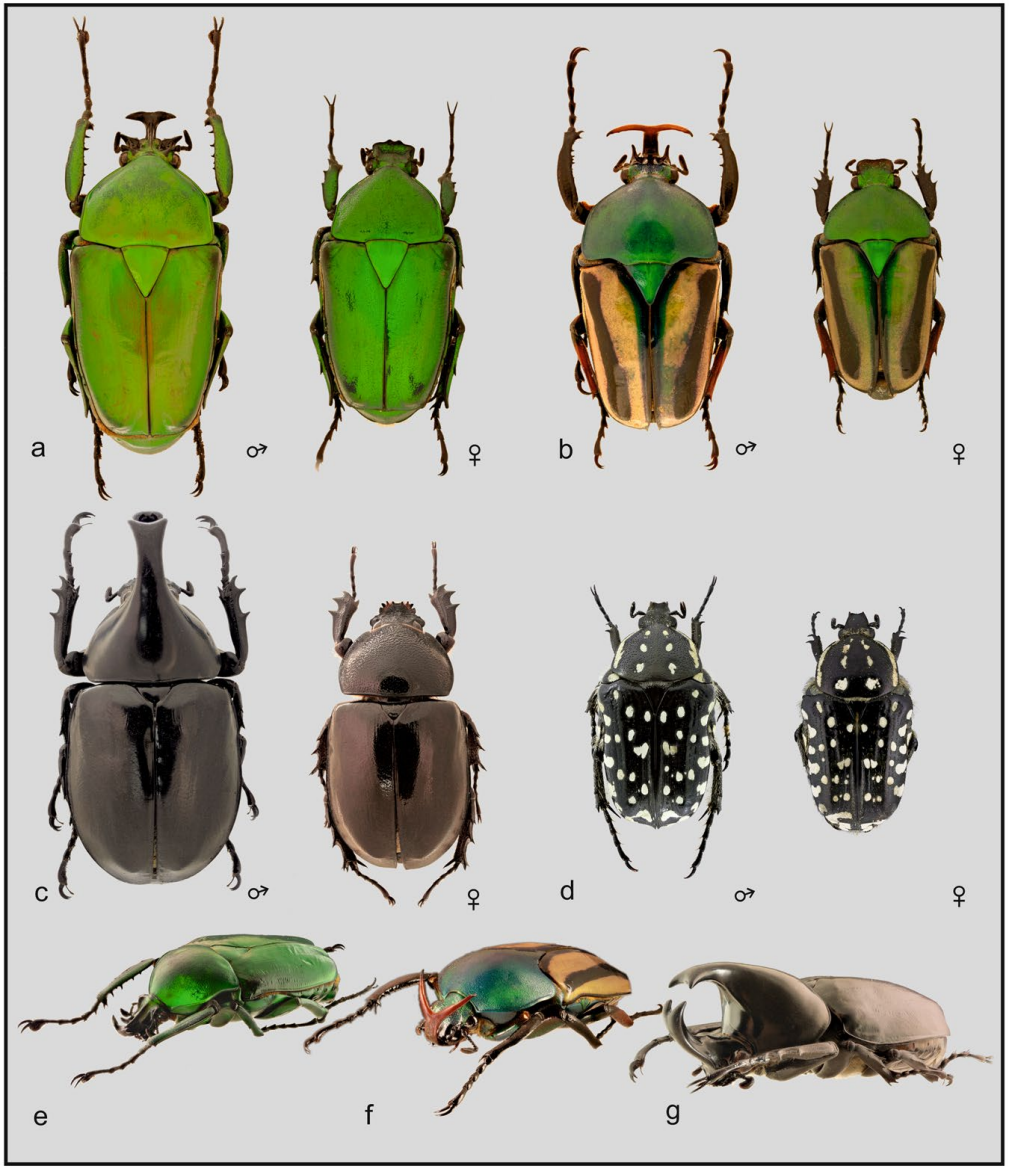
Male and female of *Dicronorhina micans* (**a**), *Eudicella gralli* (**b**) and *Xylotrupes gideon* (**c**). In *Oxythyrea pantherina* (**d**), a presence of the ventral abdominal line of white spots in males is the only external difference between sexes. Figures not to scale; the real sizes of the males and females are as follows: 49 mm and 40 mm in *D*. *micans*, 38 mm and 32 mm in *E*. *gralli*, 53 mm and 41 mm in *X*. *gideon*, and 11 mm in the case of the male and female of *O*. *pantherina*. (**e**–**g**) frontolateral views of males of the dimorphic species. (Photo of *O*. *pantherina* courtesy of D. Vondráček).

We used four species of the plant-feeding lineage of scarab beetles (Coleoptera: Scarabaeidae) as a model (Fig. 1). The striped love beetle, *E*. *gralli*, and *D*. *micans* are large cetoniins with apparent sexual dimorphism (Fig. 1 and Table 1), which is pronounced by the presence of cephalic horns and prolonged forelegs in males. Both of the species are of African origin and currently classified within the tribe Goliathini^37,38^. The rhinoceros beetle *X*. *gideon*, distributed in SE Asia and Northern Australia is similarly dimorphic, but males possess not only cephalic, but also pronotal horns. *Oxythyrea pantherina* is a small cetoniine from Northern Africa, classified within the tribe Cetoniini, without apparent dimorphism, and in this study served as a control for the highly dimorphic species.

**Table 1:** Summary of the studied species.

Species	*n* males, *n* females	Dimorphic	SSD index
*D*. *micans*	27, 26	YES	0.31
*E*. *gralli*	23, 31	YES	0.29
*X*. *gideon*	32, 29	YES	0.24
*O*. *pantherina*	25, 30	NO	−0.02

### Study design

All larvae used in this study were an F2 generation of beetles imported from nature (*D*. *micans* from Cameroon, *E*. *gralli* from Tanzania, *X*. *gideon* from Thailand, and *O*. *pantherina* from Tunisia). Adult beetles of the F1 generation were sexed and randomly paired (we established five pairs of *D*. *micans*, seven pairs of *E*. *gralli*, six pairs of *O*. *pantherina*, and six pairs of *X*. *gideon*). The pairs were kept in glass or plastic boxes of appropriate size (18 × 13 × 10 cm for *O*. *pantherina*, 30 × 18 × 30 cm for *E*. *gralli*, and 35 × 30 × 28 cm for *D*. *micans* and *X*. *gideon*). The mating boxes were filled with a mixture of soil and leaf litter and were kept at the constant temperature of 28 °C with a 12:12 L/D cycle. The beetles were fed with ripe bananas *ad libitum*. Humidity was maintained by spraying the boxes with water every 2–3 days. The substrate was checked once a week and the newly laid eggs were transferred individually into 30 ml plastic vials containing crushed beech (*Fagus sylvatica*) leaf litter. The eggs were monitored every second day to determine the date of hatching. The newly hatched larvae were weighed using a KERN 450-3 M digital scale with a precision to 0.001 g. The subsequent weighing intervals were set depending on the presumable duration of the particular instars^39,40^ and were as follows: in *D*. *micans*, every three days during the first instar, four days during the second instar, and six days during the third instar; in *E*. *gralli*, every two days during the first instar and four days during the second and the third instar; in *O*. *pantherina*, every two days during the second instar and three days during the third instar; and in *X*. *gideon*, every two days during the first instar, three days during the second instar, and seven days during the third instar. For technical reasons, these intervals could not be always observed, but the total number of replications was in all cases sufficient to fit the growth curve. The mass of the first instar was measured on average (i.e. mean number of the weight records) at 10 time points in individuals of *D*. *micans* and *X*. *gideon* and at 7 time points in *E*. *gralli*; the mass of the second instar was measured on average at 10 time points in individuals of *D*. *micans* and *E*. *gralli*, at 13 time points in *X*. *gideon* and at 6 time points in *O*. *pantherina*; for the third instar it was 21 time points in *D*. *micans*, 26 in *E*. *gralli*, 29 in *X*. *gideon* and 12 in *O*. *pantherina*. The first instar larvae of *O*. *pantherina*, owing to their mass approaching the sensitivity level of our scale (around 0.001 g), were not weighed, thus the mass at hatching is not known and only the maximal mass in the first instar and the data for second and the third instar are available for this species. In the course of their development, the larvae were successively transferred to boxes of appropriate size: the second instar larvae of *E*. *gralli* and *D*. *micans* to 250 ml boxes and third instar larvae to 500 ml boxes, the second instar larvae of *X*. *gideon* to 500 ml boxes, in which they were kept also during the third instar. Larvae of *O*. *pantherina* were kept in the same 30 ml vials during entire development. Because the growth of scarab beetles is highly influenced by food quality^41^, approximately half of the substrate was replaced with fresh substrate every weighing period to ensure optimal nutrient and moisture conditions. Boxes were kept in a climatic chamber at the average temperature of 25 °C with a 12:12 L/D cycle. In the case of *D*. *micans*, for a technical reason, the precise date of hatching was not recorded for 15 larvae. These larvae were omitted from the analysis dealing with the first instar. Estimated asymptotic size of one larva of *D*. *micans* in the second larval instar was notably outlying, much different from the measured maximal masses, probably due to an unrecorded slowdown of growth rate at the end of the instar. This individual was omitted from the analysis dealing with the second instar in this species.

For each instar, we determined developmental time, maximal mass, and average growth rate, calculated as maximal mass in the instar *n* minus maximal mass in the instar *n*−1 divided by the time spent in the instar *n*. Because the hatching mass of *O*. *pantherina* was not known, the average growth rate of the first instar was calculated as the maximal mass in the first instar divided by the instar duration. In addition to these variables, we also estimated parameters of individual growth curves (see below), namely instantaneous growth rate (i.e. actual growth rate during the period of intensive growth) and the inflection point. Body mass was measured also for adult beetles after leaving the pupal cocoon and expelling the meconium (the waste product of metabolic processes accumulated during the pupal phase). Developmental time as well as mass loss during metamorphosis (expressed as a ratio of the difference between maximal larval mass and adult mass to maximal larval mass in percents) was determined in the pupal stage.

### Data analysis

**Figure 2:**
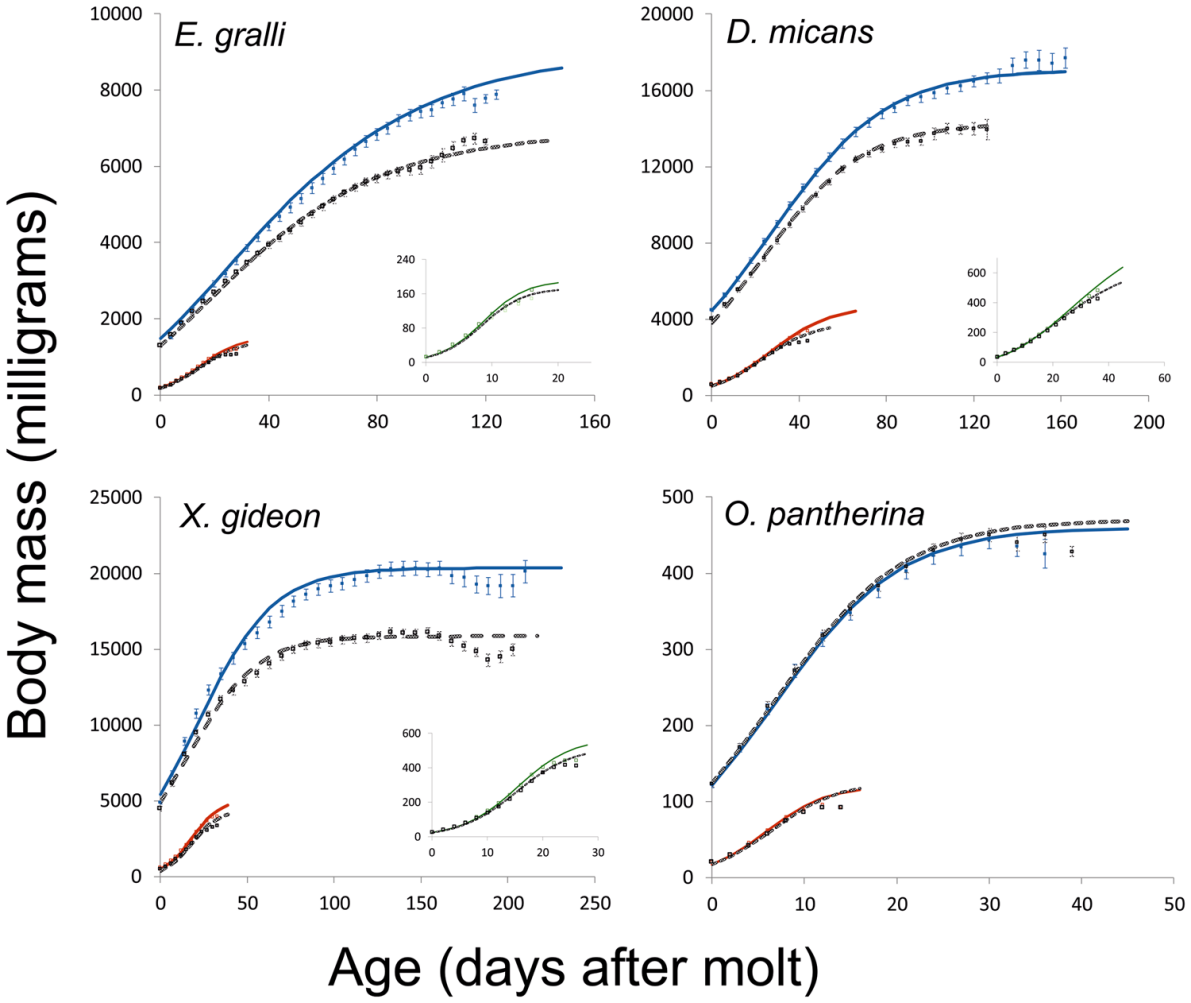
Mean growth trajectories of males (colour symbols) and females (gray symbols) of *E*. *gralli*, *D*. *micans*, *O*. *pantherina* and *X*. *gideon* within individual instars. The main pictures depict the second (red) and the third (blue) instar, the inset images display the first instar (green). Body masses are depicted as means ± SE in particular measurements. Because of certain irregularities in the weighing intervals (see text for explanation), the missing values of mass in the particular time point were replaced by estimations based on the values taken in two adjacent time points assuming linear growth in this short interval. Solid lines represent estimated Gompertz or logistic growth curves for males, dashed lines for females. Because individual larvae differ in developmental time, values of mean body mass at the end of growth trajectories do not contain larvae with distinctly shorter development.

The differences between males and females in developmental time (instar duration), average growth rate, and body mass at particular stages were tested using one-way ANOVA. Because growth in all larval instars was clearly asymptotic (Fig. 2), we fitted the asymptotic growth curves to recorded body mass separately in each individual and instar, and subsequently tested sexual differences in growth trajectories by comparing parameters of the fitted growth models in particular instars^12^. We applied three commonly used models, Gompertz^42,43^, logistic^44^, and von Bertalanffy^44,45^, to estimate the following growth parameters: the asymptotic size (*a*), the growth rate (*k*), the inflection point (*l*). In the case of von Bertalanffy model, the time when body mass is equal to 0 (T) was used instead of the inflection point. The Gompertz growth curve may be expressed as

1 $$body\,mass=a\ast {e}^{-{e}^{-k(time-l)}}$$

the logistic curve as

2 $$body\,mass=a/(1+{e}^{-k(time-l)})$$

whilst the von Bertalanffy curve as

3 $$body\,mass=a{(1-{e}^{-k(time-T)})}^{3}.$$

Because the larvae moult with an empty gut, which is refilled after ecdysis, and thus the mass increment could be attributed to the newly absorbed gut content, the first mass recording after ecdysis was not taken into account. Similarly, larval mass after gut emptying at the end of the instar was not included in the calculations of the growth models. For each instar of each species, we used parameter estimates of the model best fitting the data. Except for the first instar in *D*. *micans* and the third instar in *E*. *gralli* (where the Gompertz model was better), the logistic model fitted the data best. Overall, the von Bertalanffy model explained slightly less variation in body mass change than the other two models. In two cases (in the third instar of *E*. *gralli* and *X*. *gideon*), however, the von Bertalanffy model fitted almost identically as the other best-fitting model (99.4% and 98.3% of explained variance by both Gompertz and von Bertalanffy equation in the third instar of *E*. *gralli* and *X*. *gideon*, respectively). In these cases, owing to its easier biological interpretation, we used the Gompertz model. In all dimorphic species studied here males are the larger sex. Therefore, SSD index was expressed as the Lovich and Gibbons ratio^46^, calculated as (body mass of males/body mass of females) −1.

Because different shapes of the growth trajectories may lead to the same body size (e.g. when one sex grows faster but for a shorter period, or either grows faster or for a longer period, but finally loses a higher proportion of mass), we analyzed and intersexually compared growth also in the non-dimorphic *O*. *pantherina* to exclude the possibility that sexually dimorphic growth trajectories are a general feature in scarabs regardless of their final SSD in adults.

The variables were tested for normality using the Kolmogorov–Smirnov test and by visual inspection. When data departed from a normal distribution, non-parametric tests were used. The significance level was set to 0.05. Statistical analyses were performed using the STATISTICA programme, version 6^47^.

## Results

**Figure 3:**
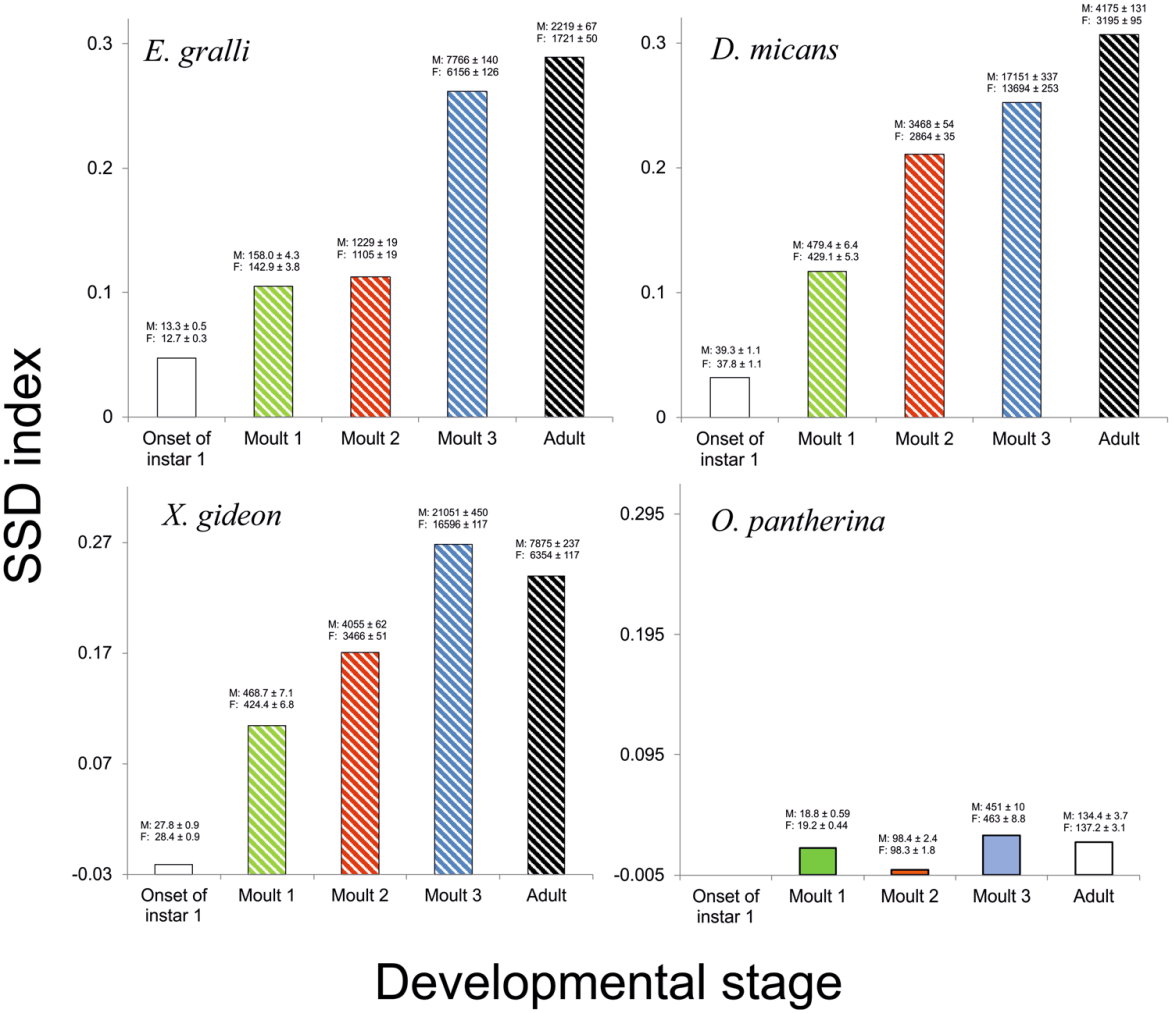
Development of SSD in four scarab species. Shaded bars indicate significant differences in body mass, blank bars indicate non-significant differences. Mean maximal body mass ± SE achieved by males and females in each instar are given above the respective bars.

We confirmed that all three tested male-larger beetle species are indeed sexually dimorphic and that *O*. *pantherina* is sexually monomorphic in body mass (Table 1). In all three dimorphic species, the dimorphism was present already early in ontogeny and accumulated through successive larval development (Fig. 3 and Table 2). There were no significant differences in hatching body mass in any of these species, but already at the end of the first instar the males were significantly larger. Finally, the males lost considerably more mass during metamorphosis in the absolute term, but the differences in relative mass loss between sexes were not significant (Supplementary Table S1), and thus the sexual dimorphism did not significantly change during metamorphosis. In *O*. *pantherina* significant SSD was not revealed at any developmental stage.

**Table 2:** Average maximal body mass (±SE) in particular developmental stages and differences between the sexes. I_in_ refers to initial body mass (i.e. the mass at hatching). Differences between sexes were tested with a one-way ANOVA.

Species	Instar	Males	Females	F	p
*D*. *micans*	I_in_	39.26 ± 1.07	37.79 ± 1.01	1.00	0.32
	I	479.4 ± 6.5	429.1 ± 5.3	36.09	≪0.001
	II	3468 ± 54	2864 ± 35	87.22	≪0.001
	III	17151 ± 337	13694 ± 253	66.42	≪0.001
	adult	4175 ± 131	3195 ± 95	36.17	≪0.001
*E*. *gralli*	I_in_	13.26 ± 0.50	12.71 ± 0.35	0.87	0.36
	I	158.0 ± 4.3	142.9 ± 3.8	6.83	0.012
	II	1229 ± 20	1105 ± 19	19.84	≪0.001
	III	7766 ± 140	6156 ± 126	72.09	≪0.001
	adult	2219 ± 67	1719 ± 52	36.83	≪0.001
*X*. *gideon*	I_in_	27.81 ± 0.94	28.41 ± 0.89	0.21	0.65
	I	468.7 ± 7.1	424.5 ± 6.7	20.16	≪0.001
	II	4055 ± 63	3466 ± 51	51.38	≪0.001
	III	21052 ± 450	16596 ± 275	68.06	≪0.001
	adult	7875 ± 237	6354 ± 117	30.94	≪0.001
*O*. *pantherina*	I	18.84 ± 0.59	19.17 ± 0.44	0.20	0.65
	II	98.44 ± 2.41	98.27 ± 1.79	0.003	0.95
	III	451.0 ± 10.0	463.6 ± 8.8	0.91	0.35
	adult	134.4 ± 3.7	137.2 ± 3.1	1.70	0.20

A higher average growth rate during the larval instars rather than longer developmental time, appeared to be the predominant mechanism allowing males to reach larger body size (Table 3). Nevertheless, because at least in the third instar the growth is highly asymptotic and there is a long period of considerably retarded growth (Fig. 2), the average growth rate is highly biased by the period of the retarded growth and it thus considerably underestimates the true growth rate^32^. For that reason, we also tested if there are any sexual differences in the instantaneous growth rate estimated from the asymptotic growth models. The applicability of these growth models on beetle growth data was supported by a relatively large proportion of explained variability (Table 4) and by the fair correspondence of the real final mass and the estimated asymptotic mass (see Fig. 2).

**Table 3:** Developmental times and average growth rates in particular instars and sexes in four scarab species. Differences between sexes were tested with a one-way ANOVA and Mann-Whitney U-test in the case of non-normal distributed data.

Species	Instar	Developmental time ± SE (days)				Growth rate ± SE (mg*day^−1^)			
		Males	Females	F (U)	p	Males	Females	F	p
*D*. *micans*	I	44.78 ± 0.37	44.04 ± 0.37	1.93	0.17	10.72 ± 0.16	9.75 ± 0.12	22.40	<0.001*
	II	48.41 ± 0.82	45.81 ± 1.08	201^(1)^	0.008^(1)^*	62.28 ± 1.63	53.66 ± 1.14	18.49	<0.001*
	III	157.93 ± 4.80	129.31 ± 4.77	17.86	<0.001*	88.10 ± 2.64	85.20 ± 2.04	0.75	0.85
	pupa	66.52 ± 1.06	65.73 ± 2.04	0.12	0.73				
*E*. *gralli*	I	19.57 ± 0.72	19.41 ± 0.67	0.14	0.88	7.57 ± 0.29	6.84 ± 0.32	2.40	0.13
	II	31.52 ± 1.03	30.24 ± 0.88	312^(1)^	0.44^(1)^	34.89 ± 1.41	32.49 ± 0.96	2.92	0.09
	III	150.52 ± 3.53	145.38 ± 4.04	0.87	0.36	43.99 ± 1.40	35.61 ± 1.37	19.14	<0.001*
	pupa	47.52 ± 0.86	47.84 ± 1.26	0.04	0.85				
*X*. *gideon*	I	27.06 ± 2.54	26.41 ± 2.64	426^(1)^	0.58^(1)^	16.44 ± 2.21	15.07 ± 1.53	7.70	0.007*
	II	35.88 ± 4.67	35.10 ± 4.64	0.42	0.52	101.32 ± 13.74	88.16 ± 14.33	13.39	0.001*
	III	228.88 ± 17.72	217.14 ± 18.02	276^(1)^	0.007^(1)^*	74.78 ± 13.03	60.77 ± 7.14	26.30	<0.001*
	pupa	34.50 ± 1.89	34.31 ± 1.93	0.005	0.94				
*O*. *pantherina*	I	13.12 ± 0.48	13.03 ± 0.33	0.02	0.88	1.48 ± 0.06	1.49 ± 0.04	0.06	0.81
	II	14.80 ± 0.72	14.73 ± 0.58	0.005	0.94	5.69 ± 0.30	5.59 ± 0.23	0.06	0.81
	III	41.80 ± 1.02	42.73 ± 0.99	0.43	0.56	8.55 ± 0.28	8.66 ± 0.24	0.08	0.78
	pupa	31.76 ± 2.73	27.80 ± 2.07	296.5^(1)^	0.19^(1)^				

^(1)^Tested by nonparametric Mann-Whitney test.

**Table 4:** Estimated growth rates (*k*) and inflection points (*l*) for all instars of four scarab species and the test of sexual differences in these parameters. Model refers to the chosen growth model according to total variance (R^2^) explained by the model; L: logistic growth curve, G: Gompertz curve. The larvae of *O*. *pantherina* have not been weighed in the first instar.

Species	Model	Instar	Sex	R^2^	*k*	F	p	*l*	F	p
*D*. *micans*	G	I	M	0.998	0.0459 ± 0.0024	6.54	0.015*	26.332 ± 1.858	7.49	0.01*
			F	0.996	0.0534 ± 0.0012			20.675 ± 0.907		
	L	II	M	0.990	0.0759 ± 0.0021	6.71	0.01*	27.631 ± 1.118	7.06	0.01*
			F	0.989	0.0851 ± 0.0029			23.321 ± 1.176		
	L	III	M	0.992	0.0380 ± 0.0013	7.56	0.008*	27.498 ± 1.353	5.4	0.03*
			F	0.993	0.0440 ± 0.0018			23.185 ± 1.364		
*E*. *gralli*	L	I	M	0.995	0.312 ± 0.018	0.04	0.85	8.716 ± 0.476	0.10	0.75
			F	0.994	0.317 ± 0.014			8.504 ± 0.447		
	L	II	M	0.992	0.135 ± 0.0074	0.04	0.84	14.605 ± 0.797	0.005	0.94
			F	0.994	0.136 ± 0.0044			14.684 ± 0.742		
	G	III	M	0.994	0.0241 ± 0.0011	4.74	0.034*	24.756 ± 1.266	15.11	<0.001*
			F	0.995	0.0273 ± 0.00094			18.840 ± 0.915		
*X*. *gideon*	L	I	M	0.993	0.201 ± 0.0046	0.12	0.73	15.426 ± 0.437	0.06	0.82
			F	0.991	0.199 ± 0.0053			15.270 ± 0.498		
	L	II	M	0.991	0.124 ± 0.0054	0.15	0.70	18.178 ± 0.833	0.001	0.97
			F	0.989	0.127 ± 0.0059			18.228 ± 1.004		
	L	III	M	0.983	0.0461 ± 0.0027	1.79	0.19	21.908 ± 0.975	20.80	<0.001*
			F	0.983	0.0503 ± 0.015			15.774 ± 0.916		
*O*. *pantherina*	L	II	M	0.992	0.306 ± 0.015	1.27	0.26	5.863 ± 0.411	1.27	0.27
			F	0.993	0.284 ± 0.012			6.549 ± 0.439		
	L	III	M	0.994	0.149 ± 0.0055	0.13	0.72	6.883 ± 0.385	0.02	0.89
			F	0.995	0.146 ± 0.0035			6.959 ± 0.384		

In contrast to a lower average growth rate, females exhibited a similar, or in the case of *D*. *micans* and *E*. *gralli* even higher *instantaneous* growth rate (parameter *k*) than males, but the period of this fast growth stopped considerably earlier in females than in males, as evident from the sexual dimorphism at the inflection point of growth trajectories (*l*; Table 4). This was true for the third instar of all three dimorphic species and also for the first and second instar in *D*. *micans*. In *O*. *pantherina*, there were no significant differences in any growth parameter in any analysed instar (Fig. 2 and Table 4).

## Discussion

We showed that significant sexual differences in body mass in three male-larger species of scarab beetles are already measurable at the end of the first larval instar, i.e. that SSD starts to develop early in their larval development. Furthermore, SSD tends to accumulate throughout the larval development. This result corresponds to the situation in Lepidoptera, where female-biased SSD is also achieved via size differences accumulating in the course of several larval instars^7^. In contrast, previous studies dealing with the ontogeny of SSD in two rose chafer species, *Mecynorhina polyphemus* and *Pachnoda marginata*, documented that males attained their larger body size within only one larval instar^12,30^. The adult males of the three male-larger species studied here are about 30% heavier than females, which is rather extraordinary among male-biased insects^5^ and probably also among rose chafers and rhinoceros beetles. On the other hand, dimorphism in body mass in *M*. *polyphemus* and *P*. *marginata* is much less pronounced, only about 10%^12,30^. These findings are congruent with the observation of Tammaru *et al*.^7^ that, in contrast to highly dimorphic species, the sexes in species with a relatively low SSD diverge in size during only one or a few instars and SSD remains constant through the rest of the development. Tammaru *et al*.^7^ suggested that growth within an instar is to a large degree constrained^48^ and therefore, the development of SSD in highly dimorphic insect species has to be split among more instars.

The SSD accumulated during the larval period can dramatically change and even disappear during metamorphosis^8,9^. We tested the possibility of a potential presence of SSD exclusively in the juvenile stage in the non-dimorphic *O*. *pantherina*. Since in this species SSD did not appear at any time during larval development, it seems that in scarabs the presence of SSD in larvae is linked to the SSD in adults. Neither in any species studied herein nor in the previous study on *P*. *marginata*^30^ there were any significant sexual differences in the loss of body mass during metamorphosis. Thus, it seems that the differential mass loss at eclosion is not responsible for the SSD observed in adult scarab beetles.

Concerning the relative contribution of average growth rate (assuming predominantly linear growth) and growth duration on SSD formation, the results are rather inconsistent. There were differences in SSD development both among species as well as among individual instars within a particular species. One could conclude that the sex-related differences in both average growth rate and the instar duration are the proximate determinants of SSD in scarab beetles. In *E*. *gralli* (this study), *M*. *polyphemus*^12^ and *P*. *marginata*^30^, males have similar instar durations as females and differ only in average growth rates, while in *D*. *micans* and *X*. *gideon* one or the other of these mechanisms plays role in individual instars, or even both together interact within a single instar (see Table 3). Sexual differences in the time spent in pupa were not recorded in any of the studied scarab species. Nevertheless, Tammaru *et al*.^7^ pointed out that many previous studies examining relative importance of growth rate and growth duration on SSD formation in insects were not adequately conducted as they mostly relied on integral recording of larval development, i.e. they calculated growth rate over a large scale (sometimes even the entire egg-to-adult) period. Such an approach may lead to incorrectly estimated growth rates resulting from assuming an incorrect allometric relationship between body size and growth rate in a situation when growth rate is not linear but changes within an instar^7^. We recorded the development of SSD in each instar more closely. We demonstrated that such a detailed approach is necessary, because larval growth within an instar is not linear but rather asymptotic (in the third instar even with a long period of highly retarded growth rate)^12,30^. We found that, in contrast to their *average* growth rate, males do not have a higher *instantaneous* growth rate than females in any instar (in fact, in several cases females are the sex with the higher growth rate). On the other hand, the males have a significantly higher inflection point of the growth curves (it holds for the final instar of all studied species plus the first and second instar of *D*. *micans*). Thus, the larger size of males is attributable to their relatively longer periods of rapid growth (in the respective instars), and not to a higher growth rate, even in the cases where the absolute instar developmental times are similar in both sexes.

Our results further confirm the importance of analyzing SSD development by means of direct, continuous recording in species with non-linear growth trajectories. Much of our current knowledge of the growth trajectory shape dimorphism comes from studies on fishes, reptiles, mammals, crustaceans, and other groups with indeterminate growth, mainly because of a need for asymptotic size as a proxy for body size, enabling body size comparisons in these taxa. In several cases from these groups, the ultimately larger sex attains its larger size not by means of higher instantaneous growth rate, but by means of extension of the period of rapid growth^49–51^. Surprisingly, there is a relatively large number of cases where the larger sex has a comparable, or even lower instantaneous growth rate relative to the smaller sex^52,53^. This is in contrast with the situation in insects, where higher growth rate was supposed to be the main mechanism by which SSD is achieved^6^, or at least where lower growth rate in the larger sex was not expected. Our results, however, indicate that this seemingly exceptional mechanism of the SSD ontogeny in insects may represent at least in some cases a methodological artifact caused by an implicit assumption of linear growth.

Most models of postnatal growth assume that SSD develops due to different solutions to a trade-off in the allocation of incoming energy to growth and body maintenance in males and females^54–56^. Moreover, sexual differences in other parameters such as the amount of food ingested, consumption efficiency, or metabolic rate have been documented to contribute to SSD development. From this energetic perspective, scarab females can slow down their growth earlier than males due to a higher investment into the developing gonads^24,33,57^, eggs or fat reserves^58,59^. The resource allocation may play also a role in the differences in growth trajectories among individual instars. Because the growth of imaginal discs, the fat body, and gonads takes place already in the larval stage in insects^33,60,61^, it is possible that resource competition between these structures and larval somatic tissues differ considerably among particular instars and that the last larval instar is the most influenced.

Previously, we revealed in the rose chafer *P*. *marginata* that the larvae purged their gut at the end of the final larval instar, then refilled their gut again, and pupated only thereafter^30^. We hypothesized that this behaviour may be related to the formation of a pupal chamber from the gut content before pupation. In support, we observed the same behaviour also in the three rose chafer species studied here, but not in the rhinoceros beetle, which does not construct a pupal chamber from the gut content, but by punning the surrounding substrate.

## Conclusions

Our data demonstrate the importance of detailed and instar-specific monitoring of growth for proper inspection of SSD development. We showed that the sexes of scarabs differ in the length of the period of fast growth even in the absence of any sexual differences in the instar duration, but also that the larger sex does not have a higher instantaneous growth rate. This role of the growth trajectory shape cannot be recognized with the commonly used ‘integral’ approach, which mistakenly identifies these situations as cases of a higher growth rate in males as the mechanism responsible for SSD formation. Such inconsistency may consequently lead to a wrong assignment of the target of selection (e.g. growth rate vs. developmental time). Lastly, our study demostrates that there are differences in growth and SSD development among individual instars; therefore, the larval development should be monitored in each instar to uncover processes under selection, reponsible for SSD. Our research on scarabs thus shows that despite their relatively long generation time, their variability makes them convenient and informative for further study of the development of SSD and growth physiology in insects.

## Electronic supplementary material

Supplementary information

Supplementary data 1
