# Supplementary material for: Hidden complexity in the ontogeny of sexual size dimorphism in male-larger beetles
Source: Sci Rep. 2018 Apr 12;8:5871. doi: 10.1038/s41598-018-24047-1 (PMC5897324; doi:10.1038/s41598-018-24047-1)
Supplement: Supplementary file 1 — Supplementary information [file 41598_2018_24047_MOESM1_ESM.pdf]

Supplementary material

Hidden complexity in the ontogeny of sexual size dimorphism in male-larger beetles

Tomáš Vendl[1*], Petr Šípek[1], Ondřej Kouklík[1] & Lukáš Kratochvíl[2]

[1]Department of Zoology, Faculty of Science, Charles University, Viničná 7, 12844 Praha 2, Czech Republic

[2]Department of Ecology, Faculty of Science, Charles University, Viničná 7, 12844 Praha 2, Czech Republic

*Corresponding author. E-mail: vendl.tomas@gmail.com

Table S1. Sexual differences in the mass loss during metamorphosis in absolute term and relatively to maximal mass in the last larval instar.

Species	Absolute mass loss ± SE (mg)			Relative mass loss ± SE (%)		
	Males	Females	p	Males	Females	p
D. micans	12975 ± 238	10499 ± 207	<< 0.001	75.8 ± 0.5	76.7 ± 0.5	0.22
E. gralli	5547 ± 91	4435 ± 82	<< 0.001	71.5 ± 0.5	72.2 ± 0.4	0.30
X. gideon	13176 ± 257	10242 ± 178	<< 0.001	62.7 ± 0.6	61.7 ± 0.3	0.16
O. pantherina	320 ± 7	327 ± 6	0.46	71.0 ± 0.3	70.6 ± 0.3	0.31
